# EnvMine: A text-mining system for the automatic extraction of contextual information

**DOI:** 10.1186/1471-2105-11-294

**Published:** 2010-06-01

**Authors:** Javier Tamames, Victor de Lorenzo

**Affiliations:** 1 Centro Nacional de Biotecnología (CNB), CSIC. C/Darwin 3, 28049 Madrid, Spain

## Abstract

**Background:**

For ecological studies, it is crucial to count on adequate descriptions of the environments and samples being studied. Such a description must be done in terms of their physicochemical characteristics, allowing a direct comparison between different environments that would be difficult to do otherwise. Also the characterization must include the precise geographical location, to make possible the study of geographical distributions and biogeographical patterns. Currently, there is no schema for annotating these environmental features, and these data have to be extracted from textual sources (published articles). So far, this had to be performed by manual inspection of the corresponding documents. To facilitate this task, we have developed EnvMine, a set of text-mining tools devoted to retrieve contextual information (physicochemical variables and geographical locations) from textual sources of any kind.

**Results:**

EnvMine is capable of retrieving the physicochemical variables cited in the text, by means of the accurate identification of their associated units of measurement. In this task, the system achieves a recall (percentage of items retrieved) of 92% with less than 1% error. Also a Bayesian classifier was tested for distinguishing parts of the text describing environmental characteristics from others dealing with, for instance, experimental settings.

Regarding the identification of geographical locations, the system takes advantage of existing databases such as GeoNames to achieve 86% recall with 92% precision. The identification of a location includes also the determination of its exact coordinates (latitude and longitude), thus allowing the calculation of distance between the individual locations.

**Conclusion:**

EnvMine is a very efficient method for extracting contextual information from different text sources, like published articles or web pages. This tool can help in determining the precise location and physicochemical variables of sampling sites, thus facilitating the performance of ecological analyses. EnvMine can also help in the development of standards for the annotation of environmental features.

## Background

One of the main objectives of microbial ecology is to address how the variations in environmental conditions can shape the composition and structure of prokaryotic communities. For this purpose, it is critical to count on accurate estimates of the composition of the prokaryotic communities, and on a precise description of the environment in study. Nowadays, the current knowledge about how the different environmental factors shape the distribution and diversity of prokaryotes is still scarce. Although the influence of some of these factors, salinity for instance, has been widely studied and discussed [1,2], the influence of many others, and especially the combination of different factors, is yet rather unknown.

A very complete ontology, EnvO, has been developed for the annotation of the environment of any organism or biological sample. There is also a smaller and easy-to-use set of terms preserving a mapping to EnvO [3] and very suitable for environmental annotation, named Habitat-Lite [4]. These tools are very valuable resources for describing environment types, but an adequate and complete definition of individual environments must be compiled in terms of their physicochemical characteristics, thus allowing the performance of direct comparisons between them. In practice the environmental samplings are often poorly characterized, and there is not a common and established protocol stating the important variables to be measured. A very promising initiative in this direction is being carried out by the Genome Standards Consortium (GSC), with the development of MIENS [5], a specification of the needed information (contextual information) to characterize environmental samplings. Contextual information is defined, according to GSC, as "The set of (meta)data describing aspects of genomes and metagenomes such as geographic location and habitat type from which the sample was taken as well as the details of the processing of a sample". GSC also develops GCDML, a Genomic Contextual Data Markup Language for annotating these data [6].

Determining the precise location of the sampling sites is also very important. It allows the linkage between environmental characteristics and geographical locations, useful to explore the existence of possible biogeographical patterns. These patterns are already well known for macro-organisms, but their existence and extent is controversial for micro-organisms [7-12]. Also, it facilitates the identification of similar sites to study the spatial or temporal variation of their compositions. For that purpose, it is not enough to specify the name of the location, but also geographical coordinates (latitude and longitude) of all or most samples must be precisely annotated.

Currently, many environmental samplings of prokaryotic composition are already available and deposited in the ENV section of the GenBank database [13]. This is a very rich source of data for studying the structure of prokaryotic communities and their relationship with the environment. More than 6.000 samples containing 16 S rDNA sequences, the most widely used phylogenetic marker, can be found there. These samples have been recently organized in EnvDB database [14]. Usually, the samples in the GenBank database are very sparsely described from the environmental point of view. The only sources of environmental information in the entries are the title and a field named "isolation_source", sometimes citing the location and the prominent characteristics of the sample site. Nevertheless, almost no environmental information can be found this way and therefore it must be looked for in the corresponding published article. If it exists, GenBank lists the corresponding PMID (PubMed Identification Number) that can be used to trace the publication describing the experiment and the sampling procedure. Then, contextual information can be extracted from it, but the manual inspection of the articles is a very tedious and error-prone process.

A large collection of text mining tools has been developed for many different purposes in the biological field. These tools have been focused mostly in applications for molecular biology and biomedicine, such as annotating genes and proteins, drugs and diseases, inferring the relationships between them and integrating that knowledge into genomic analyses [15-17]. As far as we know, no such tools have been developed for extracting information focused on ecology, although some of the techniques used in other areas can be applicable also in this context. This has been our motivation for developing EnvMine, a set of automatic text-mining tools devoted to retrieve contextual information from textual sources of any kind, EnvMine extracts physicochemical variables and geographical locations unambiguously, performing very well on both tasks. Therefore it is a useful tool for annotating ecological data and to aid in the development of annotation schemas and specifications.

## Results

### Algorithm

#### Identification of physicochemical variables

In order to avoid confusion, we think it is convenient to define the meaning of some terms used in this section, namely the 'variable', 'unit' and 'measure' terms. The process of measuring involves determining a value for a physicochemical characteristic, such as distance, temperature, or concentration. A variable is an individual physicochemical characteristic, such as distance, temperature or concentration. The result of measuring a variable is a measurement reported in some unit, which is a division of quantity (such as meter) accepted as a standard. For instance, a meter is a unit for measuring distances. And finally, a measure is the discrete value of a given variable, such as 250 (meters).

There are many physicochemical variables that can be used to characterize an environment. Some of them are widely used, such as the depth of an oceanic sampling, or the temperature of a thermal spot. Some others have been used much less frequently (for instance, the conductivity of oceanic waters, or the irrigation rate of a soil). A comprehensive list of all possible variables does not exist, and this prevents the usage of a dictionary-based approach for their identification. Also, very often the variable itself is not cited, because it is implicit in their units. For instance, "3 gr of soil were collected" (corresponding to variable "weight"), "a small primary stream drains a 1.5-ha" (variable "area"), or "the waters were at 5 degrees C" (variable "temperature"). Therefore, the system must be capable of identifying both variables and units, and of linking units to the corresponding variables unambiguously.

The first task is to create a list of units as complete as possible. Our approach to construct automatically such a list was to scan the articles for numeric values and to recover the words immediately following them, since the units are generally mentioned immediately after a measure (a numeric value). Then we crosschecked these words with a comprehensive English dictionary (UK English wordlist 1.01, http://www.bckelk.ukfsn.org/words/wlist.zip). This allowed us to eliminate all instances referring to substantives (i.e, "we repeated the experiment 10 times"). We assumed that a word following a number and not present in the dictionary must correspond to a unit (some commonly used units, such as "meter", "liter" or "kilogram", were originally in the dictionary and were removed from it). Since units can be composed of more than one word (i.e., mg liter-1 to express a concentration), we collected the longest possible string whose words fulfill that criterion. The list of units collected in this way was manually curated, to obtain a final set of 118 different units (Additional file 1, Table S1).

The second step was to effectively identify the link between the units and their corresponding variables, taking also into account that a single unit can be cited in different ways (for instance, the unit "gram" has been found written either as "gram", "gr", "grs", or "g"). This can also be done automatically by finding joint citations of variables and units. For instance, in the sentence "samples were collected at 320 mts depth", the word "mts" is a unit present in the list, and the citation of the variable "depth" implies that "mts" is a valid unit for such variable. The variable must be in the same sentence and close to the unit (five words before or after) to produce the association. We followed an iterative procedure, starting with an initial list of ten variables (temperature, size, volume, pH, concentration, time, weight, area, pressure and salinity) and adding new ones that were identified by the presence of unmatched units. The association list between variables and units obtained in this way was manually curated and completed, to obtain the correspondence of the units with 33 different variables.

In this way, it is possible to identify variables in the text even if they were not cited explicitly, simply by the presence of a numerical value (a measure) and a unit that was associated with it. Some ambiguity can exist, because some units can correspond to more than one variable. For instance, the unit "meter" denotes a linear value, which can be used to measure length, distance or depth. The complete disambiguation of these instances is only possible if the correct variable is also cited.

Some variables are expressed as a combination of units. For example, concentrations can be measured as a weight of a substance in a particular volume, which can be expressed by different combinations of units: grams/liter, gr liter-1, nanogram per milliliter, etc. The association list also includes such combinations, specifying that the arrangement of weight and volume units corresponds to a concentration. The variables can be readily identified regardless of the specific units and the notation used.

Physicochemical information can also appear in tables or, much more rarely, in figures. The information contained in tables can be extracted, because tables in HTML format can be parsed. Individual rows and columns are clearly identified by HTML tags. Extraction of physicochemical variables from the first row of the table, performed as explained above, indicate the meaning of each of the columns, and individual data points can be extracted from subsequent rows.

#### Identification of geographic locations

The retrieval of geographic locations from texts and their normalization by their geographical coordinates allows the precise determination of the location of each sampling experiment, which is critical for performing biogeographical studies. The simple retrieval of the name of the site is not enough to identify the location exactly, because a very high degree of ambiguity exists (For instance, there are almost 1.000 populated places in the world named "San Juan"). Therefore, this task can be seen as two independent issues, first identification of the putative locations, and second matching these with their precise coordinates. Some approaches for geo-disambiguation have been already proposed [18,19]

Syntactically, geographic locations are proper nouns, which are capitalized in many languages, including English. This provides a very useful morphological clue to distinguish them from common nouns. But other proper and common nouns are also capitalized (person names, brand names, chemical compounds, most acronyms). Also, the usefulness of the syntactical structure of the sentence is limited, since the geographic location can play different roles in a sentence. For instance, in the sentence "Lake Cadagno is a meromictic lake in the Piora Valley of Switzerland", "Lake Cadagno" is the subject of the sentence, whereas "Piora Valley" and "Switzerland" compose an adverbial phrase.

The approach followed to identify possible geographical names is: First, identify all noun phrases (NPs) in the sentences by the usage of a part-of-speech tagger (TreeTagger) [20]. Then, discard all NPs not containing any capitalized words, and finally crosscheck the candidate names with a list of known geographical locations. For this purpose, we have used the GeoNames database [21], an outstanding compendium of geographic names from all around the world, containing 6.5 million features plus their geographical coordinates. GeoNames also offers web services, so that it can be queried by external resources. This is a very effective method for checking whether a particular candidate name corresponds to a real geographic location. The full procedure retrieves around 87% of the original locations. An example of the division of the text in NPs and the results of submitting these to GeoNames is provided in Additional File 2, Table S2.

This method relies very much on the completeness of GeoNames database. In order to reduce this dependence on a single data source, we also set up a procedure for performing queries to Google Maps [22] in a very similar way. Google Maps also provides web services allowing the retrieval of geographical coordinates for a given query. The usage of Google Maps slightly improves the results, since the combined procedure, GeoNames plus Google Maps, allows the retrieval of 90% of the original instances.

Still, a real location cannot be identified if it is not present in any of the geographical databases used. Nevertheless, these sites often contain a reference to a geographical feature (River, Sea, Strait, Forest, etc). We have compiled a list of these features by manual curation of a list of the most common words in GeoNames database. The list contains 132 words referring to geographical features in several languages (English, Spanish, French, Portuguese and Arabic). Then, we are able to identify the capitalized nominal phrases not present in geographical databases but containing any of these words (for instance, "Ganghwa Island"). This procedure allows retrieval of an additional 3% of locations. In this case, since these locations are not present in geographic databases, no coordinates can be found for them. To overcome this probem, we are currently testing a procedure for querying Google [23] using the putative geographic name and the keywords "longitude" and "latitude". We estimate that up to 60% of the new locations could be matched with their coordinates in this way.

The second step is the disambiguation of the geographical names when more than one site is possible. This is done by inspecting the contiguous text for other locations that can help to resolve the ambiguity. Often, the name of the region, the country, or related geographical features can be found in the vicinity of the ambiguous name. For instance, cities named "Margate" exist in South Africa, England, Australia, the United States and Canada, but its citation can be easily disambiguated if it appears as "Margate, Tasmania". The procedure consists in retrieving the country or countries for all the instances of both locations, and selecting the pair of instances whose countries match. In the example above, since Tasmania belongs to Australia, the mention of Margate is also identified with the Australian city. It is not necessary that both locations be in the immediate vicinity (for instance, "Margate is a city located in the island of Tasmania"), but it is required that both be in the same sentence.

This procedure cannot directly disambiguate between two possible locations in the same country. In that case, the geographical coordinates are used to select the one with smaller distance to the location used to disambiguate. Again using the example above, if a second city named Margate exists in Australia, it would not be possible to select the correct one in the first instance. Then we would calculate the distance between the coordinates of the two cities named Margate and the coordinates of Tasmania, selecting the one with the shortest distance.

Also a problem could arise if several countries are mentioned in the same sentence, usually corresponding to lists of locations (for instance, "These cities are San Jose, Costa Rica, Kingston, Jamaica, and Caracas, Venezuela"). In practice this is a minor problem, because rarely a place with the same name is present in more than one country, and therefore there is no ambiguity (as it is the case for "Kingston"). Otherwise, the procedure assumes that the closest country in the text is the right one (as for "San Jose, Costa Rica"), which is sufficient to disambiguate most instances.

Although this procedure of disambiguation performs very well, two problems remain: first, it cannot distinguish between locations with no country attached. These names correspond mostly to undersea features (since the rest of features can be placed in at least one country), and these features are usually not ambiguous (Additional File 3, Table S3). Second, it cannot deal with instances in which no other location is cited in the same sentence. Currently, the system works by assigning the location to the most relevant place, for instance the biggest city or the most commonly cited feature. This information is automatically provided by GeoNames database, where locations are sorted by relevance. An alternative strategy is to look for country names in adjacent sentences, or even in the whole article.

#### Discriminating parts of the article

It is important to consider that most of the contextual information retrieved this way corresponds not to environmental information, but to experimental settings (in case of physicochemical variables) or providers' addresses (in case of geographical locations). Therefore, it would be very valuable to have a way to discriminate the parts of the text that describe environmental characteristics from the rest. The distinction between parts of the article according to their rhetorical contexts has been addressed by previous works, for instance by the Rethorical Structure Theory by Mann and Thompson [24], or the zone analysis approaches by Teufel and Moens [25], and Mizuta and coworkers [26].

Here, a simple approach would be to identify the sections or subsections of the articles that are most likely to contain contextual information. Examples are subsections such as "Site and sampling characteristics", "Sample collection", "Site description", "Sources of samples", etc. Nevertheless, this procedure often fails since experimental data and settings can also be found in these sections, and environmental information can appear elsewhere. Therefore, we have designed a simple classification approach for identifying individual sentences (or groups of sentences) into environmentally or experimentally-associated. We manually classified a set of 1,151 sentences into environmental (323 sentences) or experimental (828 sentences), and used the frequencies of the words in them to train several types of classifiers available in the Weka package [27]. We filtered the words using just those that were present in more than one sentence, and whose frequency in one of the sets at least doubled that in the other. In this way, we selected 607 useful words that were used to train the classifier, which was tested by means of a jack-knife cross-validation approach.

### Testing

#### Obtaining full-text articles

We used the set of environmental samplings stored in the EnvDB database as a reference set from which to extract contextual information. Since many of the sampling experiments remain unpublished and are just deposited in the database, only 36% of them can be linked to a PMID and therefore to their corresponding publication. For these, the abstract of the article is readily available, and in this way we obtained 1646 abstracts. But since contextual data are usually cited in other sections of the article (see below), the abstract is usually not enough and the retrieval of contextual information must be performed on the full-text article.

There are two main problems in accessing full-text articles. The first one is of non-technical nature: the need for a subscription to the corresponding journal. The second is that full-text articles are provided by editorials via their own web services, implying that the structure of the pages and archives can be very different between editorials and providers. It would be necesary to create a web parser capable of tracking the links to the full-texts, which could be technically complex. Fortunately, we can take advantage of the fact that all articles are identified by a Digital Object Identifier (DOI), provided by the International DOI Foundation (IDF). PubMed uses the field "AID" to store the DOI for each article (for instance, "10.1016/j.femsle.2004.11.021 [doi]"). This DOI can be feed to the IDF web server in http://dx.doi.org/, which automatically redirects to the full-text article. The queries can be constructed by merging the above address and the DOI, such as http://dx.doi.org/10.1016/j.femsle.2004.11.021, following the example above. In this way, we were able to retrieve 567 full-text articles. The article can be downloaded in several formats, but it is advisable to use xml format if available, since all information on sections and subsections of the text is preserved. Html format is also suitable. Although pdf format is usable by converting it into html or plain text, and some tools are available to perform this step, it is not practical because the format of the text is lost (including highlights such as bold letters or bigger font sizes) and therefore the headers identifying sections of the text are missed. Also problems can arise derived from the format of the text itself (for instance, several columns per page).

Sometimes, full details of the experimental procedures are provided in supplementary information. The retrieval and processing of supplementary materials is not easy: they are more difficult to retrieve since they are not identified with a DOI; the format is very variable and often they are available only as pdf files. We have not attempted to deal with this issue in the scope of this article.

#### Physicochemical variables

In order to evaluate the performance of the identification of physicochemical information, 559 instances of diverse variables were manually annotated from a set of the "Materials and Methods" sections of 15 different articles, which have not been used previously for the development of the extraction patterns. This corpus was generated by just one annotator, and therefore consistency in the annotation is ensured. This corpus is available in our web page http://brueghel.cnb.csic.es/envmine_corpuses/envmine_corpus_units.txt. Then we used EnvMine to collect measures and variables from that set. The results are shown in Table 1.

**Table 1:** Performance of EnvMine in the identification of physicochemical information.

	*Number of Instances*	*Retrieved*	*Correct*	*Recall*	*Precision*	*F-value*
Total	559	542	*526*	96.8%	97.0%	96.9%

Linked to variables	559	519	503	92.3%	96.9%	94.5%

The row "Linked to variables" refers to these instances that were linked unambiguously to a given variable. Recall is measured as the ratio between the number of correct instances and the total number of instances. Precision if the ratio between correct instances and retrieved instances. F-value combines precision and recall as their weighted harmonic mean, calculated as F = (2*P*R)/P + R.

The performance of EnvMine in the retrieval of physicochemical information is very good, achieving almost 97% recall. Only a few instances were not retrieved, corresponding mainly to concatenated measures (for instance, "-20 and -80 degrees C", " 1, 3, 5, 10, 12, and 15 mM"), or involving units that were not present in the original list (for instance, "200,000 fluorescence units"). In most cases, the retrieved instances corresponded to a real physicochemical measure, achieving a precision of 97%. The most conspicuous error affected to rotational speed measures, because these are often expressed by the "g" unit, which is confounded with "grams", and consequently with a weight measure. We have also quantified how many of these instances could not be linked unambiguously to the real physicochemical variable. This occurs just for 23 instances (4% of the total). Almost all of these correspond to size measures ("5 mm", "20 cm"), which are ambiguous since they can match lengths, diameters, distances, depths, etc. EnvMine correctly identifies these as "size" measures, but cannot disambiguate further. The errors in the identification of variables are very rare (only 3 out of 519), corresponding to instances such as "269 kg of manure ha-1", a measure of concentration that EnvMine wrongly identifies with a weight, confused by mention of "manure".

Next, we applied EnvMine to the retrieval of physicochemical variables in the set of 567 full-text articles. The results indicate that physicochemical variables can be found in all sections of the text, although they most commonly appear in the methods section of the article, where sampling and experimental settings are described (Table 2).

**Table 2:** Number of physicochemical variables found in different sections of a set of 567 full-text articles.

Section	Absolute Number	Percentage
Abstract	466	1,4%

Introduction	1202	3,5%

Materials and Methods	25139	74,1%

Results	5375	15,9%

Discussion	1718	5,1%

The most abundant variables found in that set of articles and their most common units are shown in Table 3.

**Table 3:** Physicochemical variables most cited in the set of 567 full-text articles.

Variable	Instances	Most common unit
time	9535	min

temperature	7501	degree C

concentration	6533	mM

volume	4625	liter

weight	2436	gram

pH	1177	pH

depth	849	meter

cell number	452	cells

diameter	378	mm

cell density	304	cells/ml

area	252	square meter

size	184	meter

speed	156	rpm

length	99	meter

flux	96	ml/min

pressure	80	mm Hg

radioactivity	44	Ci

distance	38	Km

rate of temperature change	34	degrees C/min

voltage	30	mV

diffusion coefficient	23	cm^2^/s

salinity	23	psu

width	21	meter

rate of concentration change	20	mol/liter day

height	20	centimeters

enzymatic specific activity	10	mol/h gr

The table shows only the variables that appear at least 10 times.

Some of these variables occur very frequently since they indicate experimental settings. Good examples are time, concentration and temperature measures. Since we are mostly interested in retrieving information about environmental characteristics, we needed to find a way to discriminate parts of the text in which these could be cited. We have trained different types of classifiers to distinguish between environmental- or experimental-associated sentences, assuming that word usage would be different and some distinctive words would appear in each. The classifiers were generated using the frequencies of words in a set of 1,151 sentences manually assigned into experimental or environmental categories, and their performance was assessed using a jackknife cross-validation test. The full results for all the tested classifiers are shown in Table 4.

**Table 4:** Results for the discrimination between environmental-or experimental-associated sentences for different classifiers in the Weka package.

*Method*	*Subset*	*Original*	*Classified*	*Correct*	*Recall*	*Precision*	*F-value*
Naive Bayes Multinomial	Exp	828	802	755	91.2%	94.1%	**92.6%**
Naive Bayes Multinomial	Env	323	349	276	85.4%	79.1%	**82.1%**

Naive Bayes	Exp	828	692	679	82.0%	98.1%	89.3%
Naive Bayes	Env	323	459	322	96.0%	70.0%	81.0%

Bayes Logistic Regression	Exp	828	796	746	90.0%	93.7%	91.8%
Bayes Logistic Regression	Env	323	355	273	84.5%	76.9%	80.5%

Bayes Net	Exp	828	617	608	73.4%	**98.5%**	84.1%
Bayes Net	Env	323	534	314	**97.2%**	58.8%	73.3%

Meta Bagging	Exp	828	752	708	85.5%	94.1%	89.6%
Meta Bagging	Env	323	399	279	86.4%	69.9%	77.3%

Rules, Decision Table	Exp	828	1041	809	**97.7%**	77.7%	86.6%
Rules, Decision Table	Env	323	110	91	28.2%	**82.7%**	42.1%

Random Forest	Exp	828	776	735	88.8%	94.7%	91.7%
Random Forest	Env	323	375	282	87.3%	75.2%	80.8%

The column "original" indicates the original distribution of sentences, "classified" shows the results of the classifier as the obtained number of sentences in each category, and "correct" specifies the number of correctly classified sentences.

The best among all the tested was a Multinomial Naïve Bayes classifier, which has an excellent performance, especially for discriminating experimental-associated sentences. These results indicate that it is possible to distinguish accurately between parts of the article.

#### Geographic locations

For testing the performance of the procedure, we manually annotated the locations found in two different sets, consisting of 50 full-text articles and 200 abstracts, respectively. As above, the corpus was generated by just one annotator, and it is available in the web address http://brueghel.cnb.csic.es/envmine_corpuses/envmine_corpus_locations.txt. We used EnvMine to retrieve geographical locations in these sets. The results are shown in Table 5.

**Table 5:** Performance of EnvMine in the retrieval of geographical locations.

*Abstracts*	*Recall*	*Precision without coordinates*	*Precision with coordinates*
Full text	86%	96%	92%

Abstracts	90%	88%	77%

Precision is measured either considering the retrieval of just the name of the place (precision without coordinates), or also the correct coordinates (precision with coordinates).

The main difference between both datasets is that in the full-text of articles many of the locations correspond to manufacturers' addresses (i.e, PE Applied Biosytems, Foster City, Calif.), which are easy to disambiguate since the state or country is commonly cited. Since this could artificially increase the precision, we performed a second test using only abstracts of articles. In both corpuses, recall and precision are rather high. Only a few locations were missed, and there are few errors, mostly consisting in the confusion of persons' names with locations. In the abstracts, the precision with coordinates is smaller because some locations were not present in the geographical databases, and were found according to the citation of a geographic feature (for example, Lake Siso, Spain). These instances were accurately retrieved (thus increasing recall), but it is not possible to obtain their coordinates.

**Figure 1:**
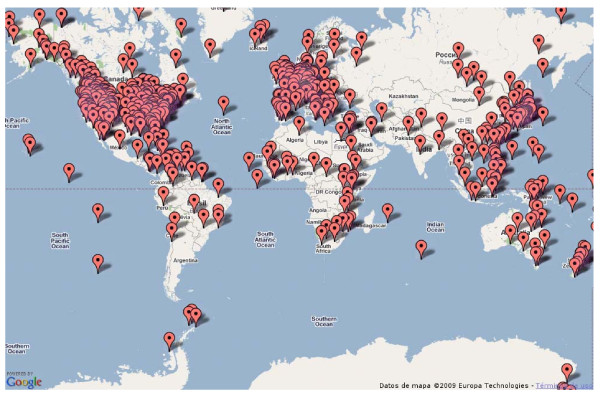
**Locations of 1646 samples stored in EnvBD database**. Original locations found in the set of 1646 abstracts corresponding to environmental samples stored in EnvDB database. 562 of these samples could be placed in the map.

Figure 1 shows the location of 562 sampling experiments from EnvDB database [28] that could be placed in the map, using just the information in the titles and abstracts of the associated publications. Although this information was very limited, EnvMine accurately retrieves the correct location when possible, including coordinates. Exceptions here are oceanic locations, which were very ambiguously cited ("Pacific Ocean", "Mid-Atlantic Ridge"), not allowing the determination of a precise location. The full-text article should be used in these cases, in the hope that more precise information can be found there. The field isolation_source of the corresponding GenBank entries also contains some information on localization, although only 365 samples could be placed in that way.

**Figure 2:**
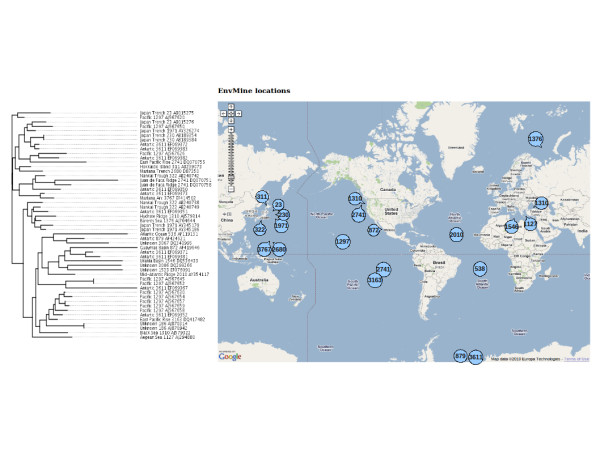
**Possible application of the retrieval of geographic locations for studying putative biogeographical patterns**. 21 samples containing a single species belonging to the archaeal *Thermoprotei *class were located using EnvMine. A phylogenetic analysis of the sequences was performed, to correlate phylogenetic distances between individual sequences and spatial distances between the samples containing them. The phylogenetic tree for the sequences is shown at the left side. Sequences' labels include the name of the location, and the EnvDB and GenBank accession numbers. The geographical locations of the samples is shown in the map at the right side.

The information on the geographic origin of the samples can be used for performing biogeographic studies such as the one shown in Figure 2. It shows the distribution of the 21 samples containing a single species belonging to the archaeal *Thermoprotei *class, also from EnvDB database. A phylogenetic analysis of the sequences has been performed, and the phylogenetic distances between individual sequences can be correlated with the spatial distances between the samples containing these sequences. In this case, a moderate correlation is obtained (Pearson correlation coefficient r = 0.41), indicating that the barriers restricting the dispersion of this particular species are weak.

## Discussion and Conclusions

We have developed EnvMine with the main purpose of extracting contextual information from scientific articles, especially those linked to sampling experiments of different environments. We have shown that the retrieval of contextual information can be performed with high effectiveness and a low rate of errors, and therefore we conclude that text mining tools like this can be very valuable in the annotation of environmental characteristics and locations. Nevertheless, we have found that such contextual information is not abundant. Very often, the samples are very sparsely described from the physicochemical point of view. Only very few variables are characterized, and there is no common criteria about which ones are to be measured. Obviously, this makes it difficult to study the influence of physicochemical factors by the comparison of results from different samples. It is clear for us that a common protocol for the collection of contextual data is needed. In this context, we find that the MIGS/MIENS standard, the initiative of the Genomic Standards Consortium to propose a minimum set of variables to be measured in each sampling, is extremely important [5]. EnvMine can help to annotate these variables from textual sources, reducing greatly the effort to produce complete descriptions of environmental samplings.

EnvMine is composed of two separated modules, devoted to the retrieval of physicochemical variables and geographical locations, respectively. Both modules can be combined to create a catalog of environmental features in different locations of the world, which can be a valuable resource for the performance of a wide range of ecological studies and in some instances could be used to annotate poorly characterized samples.

EnvMine could also be easily combined with other text-mining tools. For instance, Text Detective [29] can be used to precisely identify chemical compounds, which combined with concentration measures would provide a precise chemical profile of the environment. Recently, Spasic and colleagues described a method for constructing a controlled vocabulary in order to facilitate the retrieval of experimental techniques linked to metabolomic studies [30]. Also, Frantzi and colleagues developed TerMine, a text-mining system allowing the extraction of multi-word terms that can be used to build controlled terminology lists [31]. The combination of these tools can lead to a complete system for the extraction of experimental methodologies and protocols. This can allow the comparison of different results according to variation in the settings used in each experiment. For instance, the composition of the human intestinal microbiome is found to be rather diverse, and perhaps some apparently discordant results could be attributed to the usage of diverse experimental procedures that introduce particular biases, leading to different conclusions [32,33]. EnvMine can be used to complement the annotation of environments by EnvO or Habitat-lite.

EnvMine is part of an effort to create a comprehensive database linking prokaryotic diversity and environmental features [14,28]. We hope that this resource can help to increase our knowledge of the assembly of natural microbial communities and their relationships with the environment.

EnvMine web server can be accessed at http://brueghel.cnb.csic.es/envmine.html

## Authors' contributions

JT and VdL conceived the study. JT designed and implemented the methods, and analyzed the results. Both authors drafted the manuscript.

## Supplementary Material

Additional file 1 **Table S1**. Curated list of measurement units, their frequency in the original set of articles, and their association with physicochemical variables. Click here for file

Additional file 2 **Table S2**. Example of the NPs retrieved from the text of articles, and the results obtained when sending them to GeoNames database. NPs are shown between brackets, and these that retrieve results from GeoNames are coloured in red. The results are shown in tables below the text, including the position in the text, the type of feature and the location (disambiguated if needed), with its precise geographical coordinates. Click here for file

Additional file 3 **Table S3**. Number of instances in GeoNames database grouped by their feature type, indicating the number and percentage of ambiguous and unambiguous cases, and also the percentage of ambiguities with respect to the total. A feature is ambiguous when more than one location with identical name is present. Click here for file
