# Supplementary material for: EnvMine: A text-mining system for the automatic extraction of contextual information
Source: BMC Bioinformatics. 2010 Jun 1;11:294. doi: 10.1186/1471-2105-11-294 (PMC2901371; doi:10.1186/1471-2105-11-294)
Supplement: Additional file 2 — Table S2. Example of the NPs retrieved from the text of articles, and the results obtained when sending them to GeoNames database. NPs are shown between brackets, and these that retrieve results from GeoNames are coloured in red. The results are shown in tables below the text, including the position in the text, the type of feature and the location (disambiguated if needed), with its precise geographical coordinates. [file 1471-2105-11-294-S2.DOC]

EnvDB_ID 4 PMID 9231422

[Microbial flora] in [the deepest sea mud] of [the Mariana Trench]. In [an attempt] to characterize [the microbial flora] on [the deepest sea floor], we isolated [thousands] of [microbes] from [a mud sample] collected from [the Mariana Trench]. [The microbial flora] found at [a depth] of [10897 m] was composed of [actinomycetes], [fungi], [non- extremophilic bacteria], and [various extremophilic bacteria] such as [alkaliphiles], [thermophiles], and [psychrophiles]. [Phylogenetic analysis] of [Mariana isolates] based on [16S rDNA sequences] revealed that [a wide range] of [taxa] were represented.

RESULT

| ID | Source | Word pos | Location | Long | Lat | Type |
| --- | --- | --- | --- | --- | --- | --- |
| 4 | GeoNames | 10-11,39-40 | Mariana Trench | 147.5 | 15.0 | Undersea type feature |

EnvDB_ID 36 PMID 10589740

[Microbial diversity] in [marine sediments] from [Sagami Bay] and [Tokyo Bay], [Japan], as determined by [16S rRNA gene analysis]. [16S rDNA clone libraries] were analysed to investigate [the microbial diversity] in [marine sediments] from [Sagami Bay] ([stations SA], [water depth] of [1159 m], and [SB], [1516 m]) and [Tokyo Bay] ([station TK], [43 m]). [A total] of [197 clones] was examined by [amplified rDNA restriction analysis] ([ARDRA]) using [three four-base-specific restriction enzymes] ([Hhal], [Rsal] and [Haelll]). In [SA], [57 RFLP types] were detected from [77 clones]. In [SB], [17 RFLP types] were detected from [62 clones]. In [TK], [21 RFLP types] were detected from [58 clones].

...

[All sequences] had [>84.8% similarity] to [rDNA sequences] retrieved from [the DNA databases]. [Sequenced clones] fell into [five major lineages] of [the domain Bacteria]: [the gamma], [delta] and [epsilon Proteobacteria], [Gram-positive bacteria] and [the division Verrucomicrobia]. At [SA], [the Verrucomicrobia] and [the three subclasses] of [the Proteobacteria] were found. [Most clone sequences] belonged to [the gamma Proteobacteria]. [The high-GC Gram-positive bacteria] and [the gamma subclass] of [the Proteobacteria] were common at [both SB] and [TK]. Although [the depths] of [SB] and [TK] were [very different], [the community diversity] inferred from [ARDRA] and [the taxonomic position] of [the dominant clones] were [similar]. [All clones] belonging to [the highGC Gram-positive bacteria] collected from [both SB] and [TK] fell into [the same cluster] and are regarded as [members] of [an unknown actinomycete group]. [The clone compositions] were [different] at [each sampling site], and [clones] of [the gamma Proteobacteria] and [high-GC Gram-positive bacteria] were dominant.

RESULT

| ID | Source | Word pos | Location | Long | Lat | Type |
| --- | --- | --- | --- | --- | --- | --- |
| 36 | GeoNames | 7-8, 38-39 | Sagami Bay | 139.35 | 35.25 | Hydrograph  ic |
| 36 | GeoNames | 10-11, 57-58 | Tokyo Bay | 139.78 | 35.42 | Hydrograph  ic |
| 36 | GeoNames | 13 | Japan | 139.75 | 35.68 | Administra  tive region (Country) |
